# Adalimumab in non-infectious uveitis, towards a real-world therapeutic paradigm beyond inflammation control: A multicenter cross-sectional study

**DOI:** 10.1186/s12348-025-00529-y

**Published:** 2025-10-21

**Authors:** Carmen Antía Rodríguez-Fernández, Lorena Rodriguez-Martínez, Olalla Rodríguez Lemos, Begoña de Domingo, Pere García Bru, Antía Gestoso, Stephanie Romeo, Patricia González-Berdullas, Bahram Bodaghi, Anxo Fernández-Ferreiro

**Affiliations:** 1 FarmaCHUSLab Group, Health Research Institute of Santiago de Compostela (IDIS), Santiago de Compostela, 15706 Spain; 2 Department of Ophthalmology, Bellvitge University Hospital, L’Hospitalet de Llobregat, 08907 Spain; 3 Ophthalmology Department, University Clinical Hospital of Vigo (SERGAS), Vigo, 36213 Spain; 4 Ophthalmology Department, University Clinical Hospital of Santiago Compostela (SERGAS), Santiago de Compostela, 15706 Spain; 5 Ophthalmology Department, University Hospital Complex of Ferrol (SERGAS), Ferrol, 15405 Spain; 6 Department of Ophthalmology & Visual Sciences, IHU FOReSIGHT, APHP Sorbonne University, Paris, France; 7 Pharmacy Department, University Clinical Hospital of Santiago de Compostela (SERGAS), Santiago de Compostela, 15706 Spain

**Keywords:** Adalimumab, Anti‑TNF‑alpha, Non infectious uveitis, Intraocular inflammation, Real life experience, Trough levels, Biosimilars, Patient reported outcomes, Personalized medicine

## Abstract

**Background:**

To evaluate the real-world long-term efficacy and safety of adalimumab (ADA) in refractory non-infectious uveitis (NIU), analyzing clinical outcomes, treatment adjustments, and the potential role of ADA trough levels (ADA TL) in therapeutic response. By identifying factors influencing response, our study aims to contribute to a more personalized approach.

**Methods:**

A multicenter, cross-sectional, observational study was conducted in NIU patients receiving ADA for ≥ 6 months. Disease activity was defined using the Standardization of Uveitis Nomenclature (SUN) criteria, and patients were categorized as responders (R) or non-responders (NR). The study assessed corticosteroid (CS) and immunosuppressive therapy adjustments, relapse rates, adverse events (AEs), patient-reported outcomes (PROMs), and the relationship between serum ADA TL, anti-drug antibodies (AAA), and clinical response.

**Results:**

A total of 52 patients (91 eyes) with a median disease duration of 6.5 years (IQR 3.0–11.5) were included. Anterior uveitis was the most frequent subtype (34.6%), followed by panuveitis (26.9%), posterior (25%) and intermediate uveitis (13.5%). ADA therapy resulted in a significant reduction in systemic CS and c-DMADs. At the study visit, 65.4% of patients achieved complete inflammatory quiescence, with anterior (OR = 0.06, *P* = 0.009) and posterior uveitis (OR = 0.11, *P* = 0.04), being associated with a higher likelihood of response. ADA TL and AAA did not correlate with clinical response (median [IQR]: *R* = 8.5 [3.9–14.8] µg/mL vs. NR = 10.3 [7.9–14.5] µg/mL). AEs were reported by 32.7% of patients, predominantly mild-to-moderate infections, fatigue, and headaches. No significant differences in efficacy, safety, or relapse rates were observed between original ADA and biosimilars.

**Conclusions:**

Original ADA and biosimilars demonstrate long-term effectiveness and safety in NIU, reducing the need for systemic therapy, with comparable effectiveness and safety. PROMs further revealed that improvements in vision-related QoL were primarily driven by general vision perception, underscoring the subjective impact of treatment beyond objective inflammation control. While serum ADA levels were influenced by some clinical variables, they did not correlate with response to treatment.

## Introduction

Uveitis encompasses a heterogeneous group of diseases with over 30 different phenotypes and diverse etiologies, all characterized by intraocular inflammation [1]. It is typically classified as either infectious or non-infectious uveitis (NIU), and the Standardization of Uveitis Nomenclature (SUN) working group further classifies uveitis by onset, anatomical location, course, duration, and activity [2]. This variability results in distinct patterns with diagnostic, prognostic, and therapeutic implications. However, the goal of treatment remains the same in all cases: to control ocular inflammation. If uveitis is not treated properly, persistent ocular inflammation can lead to severe visual impairment, accounting for 10–15% of blindness cases in developed countries and ranking it as one of the primary causes of blindness globally [3]. Therefore, effective treatment is required to minimize visual loss, while avoiding treatment-related toxicity.

The current standard of care for NIU is corticosteroids (CS). However, the long-term use of moderate to high doses poses risks of adverse effects, including both ocular and systemic complications. The advent of biologic agents offers faster therapeutic benefits, steroid-sparing potential, and improved safety profiles compared to conventional immunosuppressants [4]. To date, adalimumab (ADA), a monoclonal antibody targeting tumor necrosis factor-alpha (TNF-α), stands out as the only biologic approved by the FDA and EMA for non-infectious intermediate, posterior, and panuveitis [5]. The FDA’s approval of ADA for NIU in 2016, based on the VISUAL trials [5], marked a new “biological era”. Since the original ADA patent (Humira^®^) expired in 2018, multiple biosimilars were approved offering potential for cost savings and broader access [6].

The primary objectives of NIU treatment are to control inflammation, induce disease quiescence, prevent relapses, and preserve optimal visual function. Several factors can influence treatment response of biologics, among which plasma drug levels. Therapeutic ranges for ADA have been established in immune-mediated diseases such as inflammatory bowel disease (IBD) [7], psoriasis [8], rheumatoid arthritis (RA) [9], and juvenile idiopathic arthritis (JIA) [10], but they remain undefined for NIU. Current evidence regarding the relationship between ADA trough levels (TLs) and clinical outcomes in NIU is scarce and controversial. For example, while higher ADA TL have been linked to better clinical outcomes in some studies [11, 12], recent findings by Yuan et al. [13] suggest that ADA TLs may not correlate with ocular inflammation recurrence. Moreover, immunogenicity remains a significant challenge with biologics, including ADA, as it can lead to the production of anti-drug antibodies (AAAs), reducing therapeutic levels and limiting efficacy [11].

The low prevalence of NIU limits real-world studies and few retrospective works have shown ADA’s effectiveness in reducing ocular inflammation [8–10]. Even fewer studies have assessed its impact on patients’ perceived quality of life, despite increasing recommendations from regulatory agencies to incorporate Patient-Reported Outcome Measures (PROMs) and Patient-Reported Experience Measures (PREMs) into clinical research and routine practice. Visual impairment often reduces patients’ health-related and vision-related quality of life (HRQoL and VRQoL) [14] highlighting the broader impact of uveitis on patients’ daily lives beyond clinical measures. To this end, validated tools like the National Eye Institute Visual Function Questionnaire 25 (NEI VFQ-25) [15] and the European Quality of Life-5 Dimensions (EQ-5D) have been widely used [14]. The NEI VFQ-25, derived from the 51-item NEI-VFQ [15], specifically evaluates visual function, while the EQ-5D is a standardized measure for overall health outcomes. A post hoc analysis of the VISUAL-1 and VISUAL-2 trials [16] indicated that ADA might improve VRQoL in patients with NIU compared to placebo, although these controlled findings may not fully reflect real-life settings. A recent real-world study further supported these improvements in QoL and healthcare utilization with ADA [17]; however, its observational nature and relatively short follow-up (12 months) limit the ability to draw long-term conclusions. Additionally, the study focused on active NIU cases, leaving open questions regarding ADA’s impact on stable or long-term responders.

While previous studies have explored the efficacy of ADA in NIU, gaps remain regarding its long-term real-world impact, particularly in relation to treatment response predictors for personalized therapeutic strategies over time, especially during treatment tapering. Further investigation is needed to determine whether ADA TL could aid in therapeutic decision-making and how treatment adjustments influence sustained disease control. This study aims to provide real-world insights into these aspects by analyzing clinical outcomes, QoL metrics, and the relationship between ADA TL and treatment response in a heterogeneous NIU population under long-term ADA therapy. We aim to contribute to a more personalized NIU management approach by integrating real-world clinical, QoL, and pharmacokinetic insights.

## Methods

### Patients and Study Design

A multicenter, observational, cross-sectional study was conducted across four Spanish tertiary-level hospitals: University Clinical Hospital of Santiago de Compostela, University Clinical Hospital of Vigo, Bellvitge University Hospital and University Clinical Hospital of Ferrol. This study focused on patients with NIU who were treated with ADA as a therapeutic option for refractory uveitis. The study protocol was approved by the Galician Ethics Regional Committee with the registration code 2021/014 and the study was conducted under the principles of the Declaration of Helsinki and according to the Spanish Law of Biomedical Research no. 14/2007. Patients were evaluated for the study assessment between July 2022 and August 2023.

Inclusion criteria: Patients diagnosed with NIU requiring ADA to control intraocular inflammation, with a minimum treatment duration of 6 months. All patients included were above 2 years of age and signed the written informed consent voluntarily. Subcutaneous ADA treatment was initiated due to inefficacy of first-line therapy, defined as the persistence of inflammation despite the use of the highest tolerated dose of corticosteroids (CS), Disease-Modifying Antirheumatic Drugs (DMARDs) or their combination, or the presence of recurrences above a dosage threshold.

Exclusion criteria: intraocular surgery within the previous 3 months, prior treatment with another biologic agent without an adequate washout period (at least five half-lives), severe non-proliferative or proliferative diabetic retinopathy, clinically significant diabetic macular edema or other cause, age-related macular degeneration, pregnancy or lactation, incomplete ocular examinations or low transparency of optical media.

The management of the patients was conducted collaboratively by ophthalmologists, rheumatologists, pharmacists and internists within a multidisciplinary team. The diagnosis of NIU was based on clinical symptoms, ophthalmological examination, and routine biochemical, immunological, and hematological analyses. Medical visits for examination and sample collection were scheduled according to the patient’s ADA dosing calendar, allowing for a time window of 72 to 0 h before the next administration.

### Variables to study

####  Clinical characteristics

Patients’ characteristics were recorded including age, sex, weight, height, and smoking habit, as well as presence of associated systemic disease to NIU or other comorbidities.

Uveitis etiology and characteristics, including laterality (alternating unilateral or sequential involvement considered as “bilateral”) and anatomic localization, were classified according to the Standardization of Uveitis Nomenclature (SUN) Working Group criteria [2].

Eye clinical examination included best corrected visual acuity (BCVA), anterior chamber cells (ACC) and vitreous haze grading (VH), presence of snowballs/snowbanks, papillitis, perivascular sheathing, inflammatory retinochoroidal lesions, macular edema (ME), epiretinal membrane (ERM) or macular neovascularization (MNV). BCVA was determined for each eye separately with appropriate corrective lenses based on patients’ current refraction and was converted to logarithm of the minimum angle of resolution (logMAR) [2] for analysis. The presence of papillitis, vascular abnormalities and/or inflammatory retinochoroidal lesions was determined by indirect ophthalmoscopy under pharmacological mydriasis, fundus autofluorescence and angiography (if needed). The presence of ME, ERM or MNV was based on optical coherence tomography (OCT).

Data regarding past and current concomitant treatments received for NIU including information related to treatment modifications since ADA initiation was also recorded. The ADA regimen was initially prescribed every other week, per uveitis guidelines. Over time, intervals were adjusted at the clinicians’ discretion: standard (every 15 days), intensified (weekly), or extended (≥ 3 weeks). Uveitis relapses occurring after ADA initiation were recorded according to clinical charts. Additionally, patient-reported safety events were recorded to monitor the safety profile of ADA. Clinical and demographic data were managed using the electronic system Research Electronic Data Capture (REDCap) [18].

#### Disease activity evaluation: responders and non-responders to ADA

Severity of the inflammation was classified according to the SUN criteria [2]. ACC were graded through slit lamp biomicroscopy examination (within a 1 × 1-mm slit beam) before the instillation of mydriatic eye drops from 0 to 4 + in an ordinal scale [2]. Vitreous haziness was measured through indirect ophthalmoscopy after mydriasis and graded from 0 to 4 + using a standardized photographic scale according to the Nussenblatt criteria [19] as described in the National Eye Institute Criteria (NEI) adapted by the SUN Working Group [2]. Uveitis was classified as active with an ACC grade of 0.5 + or higher, a vitreous haze grade of 1 + or higher, papillitis, vasculitis, vitreous or pars plana infiltrates, or at least one active inflammatory chorioretinal or retinal vascular lesion, with multimodal imaging incorporated into the assessment [2].

Classification of patients based on response to ADA was performed at a single cross-sectional study visit. Patients with unilateral involvement who showed no signs of active inflammation on the day of the visit were classified as responders, while those with signs of inflammation were classified as non-responders. In bilateral cases where one eye had no signs of inflammation and the other showed active inflammation (*n* = 4), the eye with active disease was considered, and the patient was classified as a non-responder. Patients were also classified as non-responders when both eyes showed inflammatory signs (*n* = 17), with the most affected eye defined by the highest ACC, highest VH, and lowest BCVA (logMAR). If neither eye showed active inflammation (*n* = 18), the patient was considered a responder, using the right eye as the reference.

####  Patient-Reported outcomes

In parallel to the clinical evaluation, patients completed the 25-item version of the NEI VFQ-25 and the EQ-5D. The former consists of 25 questions grouped into 12 subscales, one related to the general health and the others to vision-related concerns. The scores obtained in each subscale are converted into a score ranging from 0 (worst vision-specific quality of life) to 100 (best vision-specific quality of life). The final NEI VFQ-25 score is the average of all items (except for the general health item) [15]. For the EQ-5D, participants are asked to assess their level of function by answering questions related to mobility, self-care, usual activities, pain/discomfort, and anxiety/depression [14].

####  Adalimumab trough levels

Serum samples for determination of ADA trough levels (TL) and anti-adalimumab antibodies (AAA) were collected maximum 72 h before the next dose of ADA administration, centrifuged and frozen until shipped to the laboratory. ADA TL and AAA were determined as previously described [20]. Briefly, ADA TL (µg/mL) were measured using the Promonitor^®^-ADL kit (#5080230000, Progenika Biopharma, Grifols, Derio, Spain) on the Titrius semiautomated ELISA platform (Grifols). AAA were determined semi quantitatively with the Quantum Blue^®^ (QB) lateral flow immunoassay on the Quantum Blue Reader (Bühlmann Laboratories, Schönenbuch, Switzerland). AAA were expressed as positive or negative using the cut-off for positivity defined by the manufacturer. All measurements were performed in parallel.

Additionally, ADA trough levels were assessed using the Quantum Blue^®^ lateral flow immunoassay. A comparative analysis was conducted between both techniques to ensure consistency in their interpretation and to confirm that no significant discrepancies were observed in ADA quantification across methods.

####  Statistical analysis

Descriptive statistics of the continuous variables are shown as the mean ± standard deviation (SD) or the median with the interquartile range (IQR). For dichotomous variables, the frequency with the corresponding percentage is provided. Continuous variables were compared with the Mann–Whitney U test, whereas the χ^2^ test or the Fisher’s exact test was used to compare dichotomous ones. Correlation analysis was performed with the Spearman rank’s correlation coefficient. The association between clinical variables and treatment response to ADA was assessed with logistic regression analysis. p-values below 0.05 were considered statistically significant. Data analysis was performed using Statistica software (StatSoft, Tulsa, OK, USA) and graphs were made using Graph Pad Prism^®^ v.9.0.1 software (GraphPad Software, San Diego, CA, USA).

## Results

### Patients’ demographics, NIU characteristics and eye clinical examination

A total of 91 eyes of 52 patients were recruited. The median age was 46.5 years, with 53.5% of the patients being female. Age at NIU diagnosis was 36.0 years, with a median uveitis duration of 6.5 years. The most prevalent location of uveitis was anterior (34.6%), followed by panuveitis (26.9%), posterior (25%), and intermediate (13.5%), with bilateral involvement in 75% of cases. Regarding the etiology of ocular inflammation, 20 patients (38.5%) were classified as idiopathic, 22 patients (42.3%) had an immune-mediated systemic disease related to uveitis, and 10 patients (19.2%) were classified with a primary ocular syndrome. More detailed information is shown on Table 1.

**Table 1:** Demographics, clinical characteristics and ophthalmologic examination of NIU patients at the time of study

Patient demographics and characteristics of NIU	*n* (%) or median (IQR)
**Hospital**	
• University Clinical Hospital of Vigo	24 (46.2)
• University Clinical Hospital of Santiago de Compostela	17 (32.7)
• Bellvitge University Hospital	8 (15.4)
• University Clinical Hospital of Ferrol	3 (5.8)
Sex (female/male)	28/24 (53.8/46.2)
Age (years)	46.5 (23.5–56.0)
**BMI**	23.5 (21.3–29.1)
• Healthy weight (18.5–24.9)	30 (58.8)
• Overweight (25-29.9)	15 (29.4)
• Obesity (≥ 30)	6 (11.8)
Age at NIU diagnosis (years)	36.0 (16.5–45.5)
Uveitis duration (years)	6.5 (3.0-11.5)
**Location of NIU**	
• Anterior	18 (34.6)
• Intermediate	7 (13.5)
• Posterior	13 (25.0)
• Panuveitis	14 (26.9)
Laterality (unilateral/bilateral)	13/39 (25.0/75.0)
Granulomatous NIU (yes/no)	2/50 (3.8/96.2)
**Etiology of NIU**	
Idiopathic	20 (38.5)
• Panuveitis	7 (13.5)
• Recurrent anterior uveitis	5 (9.6)
• Pars Planitis	5 (9.6)
• Intermediate	1 (1.9)
• Posterior	1 (1.9)
• Vasculitis	1 (1.9)
Associated to immune-mediated systemic disease	22 (42.3)
• Ankylosing spondylitis/HLAB27+	10 (19.2)
• Behçet	6 (11.5)
• AIJ	2 (3.8)
• Sarcoidosis	2 (3.8)
• Psoriasis	1 (1.9)
• Vogt-Koyanagi-Harada disease	1 (1.9)
Ocular primary syndromes	10 (19.2)
• Multifocal choroiditis	4 (7.7)
• Birdshot chorioretinopathy	3 (5.8)
• Serpiginous choroiditis	3 (5.8)
**Disease evaluation (affected eyes, ** ***n*** ** = 91)**	
• Visual Acuity (LogMAR), mean (SD)	0.3 (0.66)
• Intraocular pressure, mean (SD)	13.4 (2.7)
• Synechiae	18 (19.8)
• Macular edema	4 (4.4)
• Central subfield thickness (µm)	235.0 (186.0-278.0)
• Snowballs	12 (13.2)
• Snowbanks	4 (4.4)
• Epirretinal membrane	19 (20.9)
• Papillitis	5 (5.5)
• Active Chorioretinal lesions	1 (1.1)
• Macular neovascular membrane (Inactive/Active)	4/0 (4.4/0)

### Clinical effectiveness and patient reported outcomes

ADA demonstrated efficacy in the treatment of NIU by achieving better control of intraocular inflammation. At the time of the study, 34 patients (65.4%) showed no signs of intraocular inflammation and were classified as responders. In contrast, 18 patients classified as non-responders exhibited some signs of inflammation. Upon further analysis of this subgroup, 11 patients (21.2%) had only 0.5 + ACC, while 7 patients (13.5%) had at least 1 + activity in anterior chamber or vitreous.

We found that the response to ADA varied significantly depending on the anatomical location of uveitis. Anterior uveitis (OR = 0.06, 95% CI = 0.007–0.52, *P* = 0.009) and posterior uveitis (OR = 0.11, 95% CI = 0.01–0.96, *P* = 0.04) were associated with a higher likelihood of clinical response. In contrast, intermediate uveitis (OR = 16.5, 95% CI = 1.70-160.16, *P* = 0.01) and panuveitis (OR = 9.4, 95% CI = 2.24–39.26, *P* = 0.002) were associated with an increased probability of non-response.

**Fig. 1:**
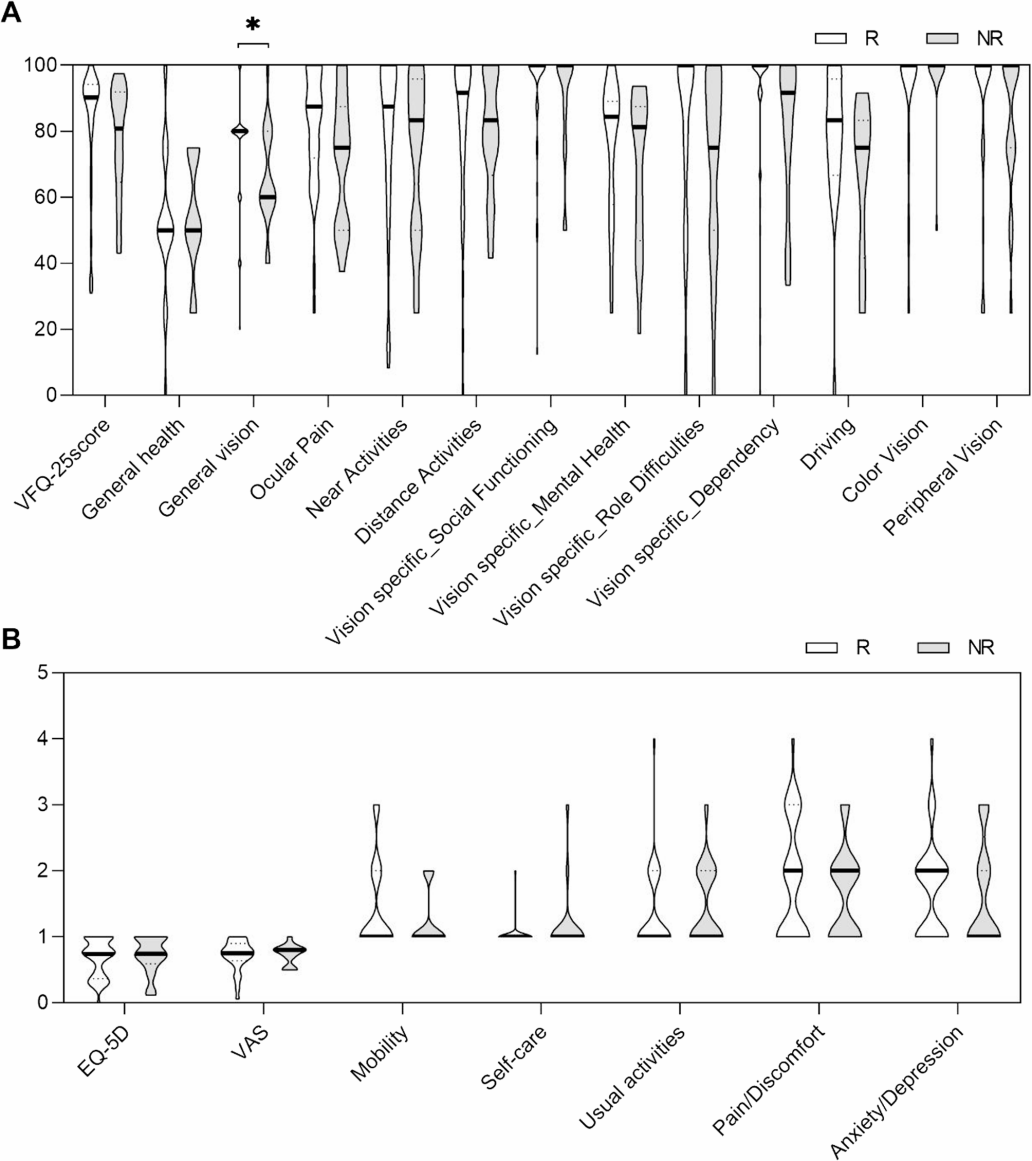
Comparison of Patient-Reported Outcome Measures between R and NR to ADA treatment. **A **VFQ-25 and subscales. **B** EQ-5D and dimensions. The width of each curve corresponds with the approximate frequency of data points in each region, with wider sections representing more frequent values, and narrower sections less frequent values. Bold line represents the median whereas point lines represent the IQR. VFQ-25: National Eye Institute Visual Function Questionnaire with 25 items, EQ-5D: EuroQol-5 dimensions-5 levels questionnaire, VAS: visual analogue scale

We then explored whether PROMs related to visual function and general health could reflect differences in treatment response to ADA. In our cohort, NEI VFQ-25 and EQ-5D overall scores were identical (Fig. 1), although a detailed analysis of the NEI VFQ-25 showed that R had a better General vision outcome than NR (Fig. 1A, *P = 0.03).

###  Pharmacological modulation of treatment

The median time since NIU diagnosis to ADA initiation was 28.0 months, and patients had been on ADA treatment for duration of 43.5 months. Before starting ADA, all patients had received corticosteroids (CS), conventional synthetic disease-modifying antirheumatic drugs (c-DMARDs) (88.5%), a different biologic drug (11.5%), or a combination of at least two drug classes (98.1%). The most common prior treatments were oral CS (86.5%), topical CS (67.3%) and methotrexate (MTX) (61.5%), followed by cyclosporine (19.2%). The maximum dose of oral CS was 50 mg/day (IQR 30–60) before ADA initiation.

Most patients (84.6%) adhered to the standard regimen every other week, with original ADA (Humira^®^) being the most used brand (57.7%) (Table 2). NIU patients on Humira and biosimilar subgroups showed a similar response rate (70.0% vs. 59.1%). Response rates by ADA dosing regimen were as follows: every-other-week (*n* = 44, 65.9% responders), weekly (*n* = 3, 0% responders), and extended interval ≥ 3 weeks (*n* = 5, 100% responders).

**Table 2:** Treatment adjustment before ADA and at the time of the study

	*n* (%) or median (IQR)
**Treatment before ADA initiation**	
Combination (CS ± c-DMARD ± Biologic)	51 (98.1)
Corticosteroids	52 (100.0)
Time with oral CS (months)	10.0 (2.0-39.5)
c-DMARDs	46 (88.5)
• Methotrexate	32 (61.5)
• Mycophenolate	5 (9.6)
• Sulfasalazine	8 (15.4)
• Cyclosporine	10 (19.2)
• Azathioprine	5 (9.6)
• Colchicine	3 (5.8)
• Hydroxychloroquine	1 (1.9)
Biologic agent (b-DMARDs)	6 (11.5)
• Etanercept	4 (7.7)
• Infliximab	1 (1.9)
• Tocilizumab	1 (1.9)
**Concomitant therapy at the study visit**	
Corticosteroids	17 (32.7)
Time with simultaneous oral CS and ADA (months)	4.0 (0–22.0)
c-DMARDs	31 (59.6)
• Methotrexate	20 (28.5)
• Mycophenolate	4 (7.7)
• Sulfasalazine	4 (7.7)
• Cyclosporine	5 (9.6)
• Azathioprine	3 (5.8)
• Colchicine	2 (3.8)
Time with simultaneous c-DMARD and ADA (months)	18 (0–40)
ADA therapy	
Response to treatment (yes/no)	34 (65.4)/18 (34.6)
Posology	
• Standard (every other week)	44 (84.6)
• Intensified (weekly)	3 (5.8)
• Reduced (≥ 3 weeks)	5 (9.6)
Dosage	
• 40 mg/20 mg	51 (98.1)/1 (1.9)
Age at ADA initiation (years)	40.0 (18.8–50.8)
ADA treatment duration (months)	43.5 (23.0–79.0)
Time since NIU diagnosis to ADA initiation (months)	28.0 (14.3–82.3)
Commercial name of ADA	
• Amgevita	2 (3.8)
• Humira	30 (57.7)
• Hyrimoz	13 (25.0)
• Imraldi	7 (13.5)

**Fig. 2:**
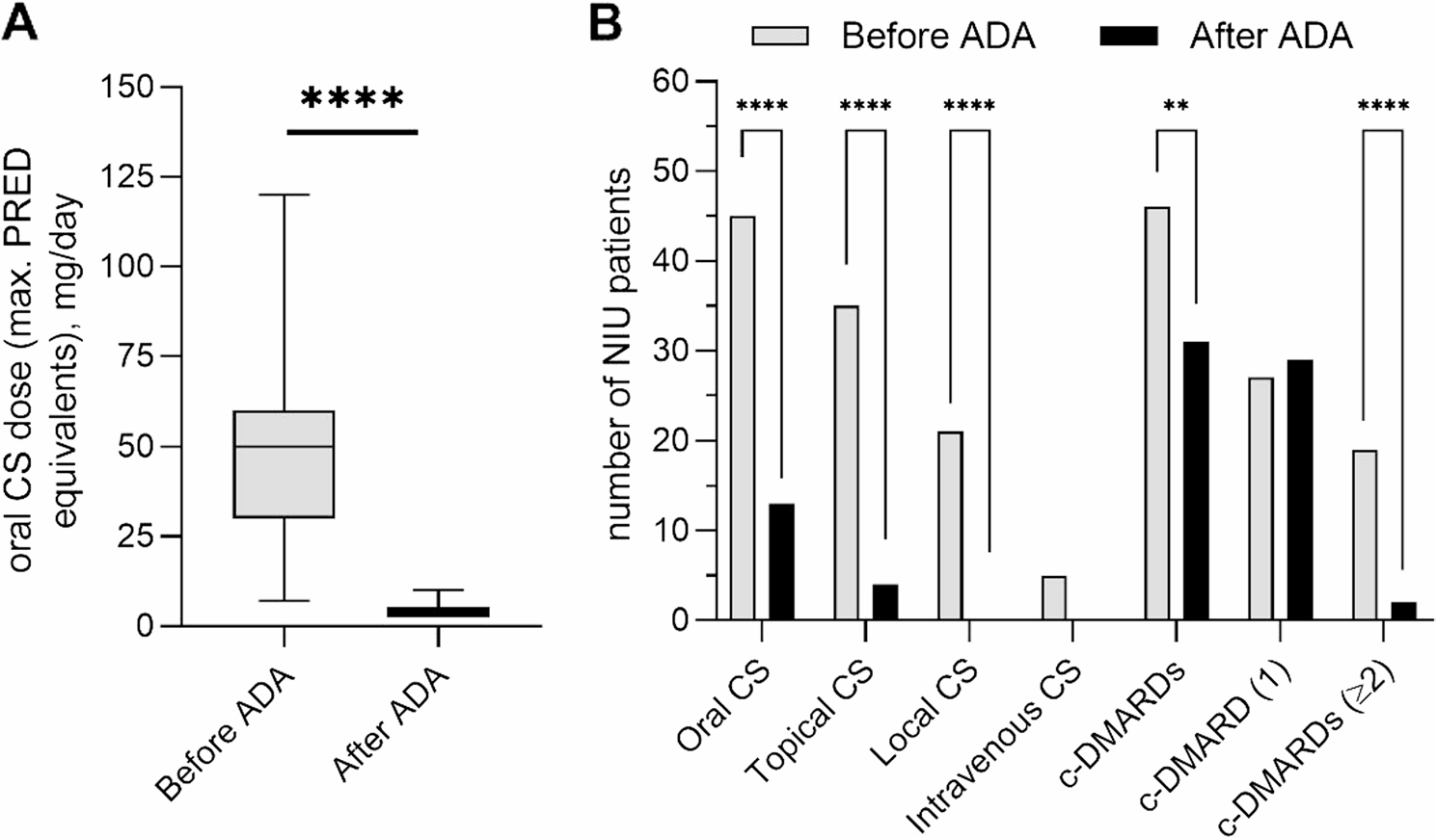
Therapy with CS and c-DMARD in patients with NIU before ADA initiation and at the time of the study. **A **Levels of maximum dose of oral corticoids expressed as prednisone equivalents before and after ADA treatment. **B** Number of NIU patients on CS or c-DMARD agents before and after ADA treatment. (* *p* < 0.05, ** *p* < 0.01, *** *p* < 0.0001)

ADA therapy significantly reduced oral CS use (*p* < 0.0001), lowering its use from 86.5 to 25% of patients, and allowed dose tapering to a median of 5 mg/day (IQR: 2.5-5.0) prednisone equivalents, Similarly, reductions were observed in c-DMARDs (*p* < 0.01) use (Fig. 2). NR patients received simultaneous CS and ADA for a longer duration (median [IQR] = 12.50 [0.75–48.25] months) than R (median [IQR] = 0 [0-14.25] months, *P* = 0.01). However, no significant differences were observed in c-DMARDs dosage or treatment duration between both groups.

During ADA treatment, half of the patients (50%) experienced relapses or flares. However, the flare rate relative to ADA treatment duration was low (median [IQR] = 0 [0–0.2] flares/month). Among those who relapsed, 61.5% had only one flare, and in 60% of cases the inflammation was limited to the anterior chamber. The overall relapse rate was comparable between R and NR, but significant differences were found in the location and pattern of the relapse: NR had significantly more intermediate (40.0% vs. 0%, *p* = 6.0 × 10^−4^) and bilateral relapses (65.0% vs. 8.0%, *p* = 1.0 × 10^−4^), while R had more anterior (84.0% vs. 30.0%, *p* = 5.0 × 10^−4^) and unilateral involvement.

In terms of safety, the side effect profile of ADA was acceptable (Table 3). Seventeen patients (32.7%) reported the presence of any AE. The most common were infections, fatigue and headaches. None of the reported AEs led to hospitalization, and most of them were of mild-to-moderate intensity (70.8%) with a median duration of 7.0 days (IQR 2.0–30.0). No differences were observed in either the frequency of AEs or their intensity between humira and biosimilars.

**Table 3:** Parameters related to the safety profile of ADA

ADA safety	*n* (%) or median (IQR)
Presence of self-reported AEs related to ADA (*n* = 52)	17 (32.7)
One	11 (21.2)
Two	5 (9.6)
Three	1 (1.9)
Type of AE (*n* = 52)	
• Infection	7 (13.5)
• Fatigue	6 (11.5)
• Headache	5 (9.6)
• Local swelling/hematoma at the injection site	2 (3.9)
• Abdominal pain and weight loss	1 (1.9)
• Lower limbs discomfort	1 (1.9)
• Erectile dysfunction	1 (1.9)
• Depression	1 (1.9)

### Adalimumab trough levels

In our cohort, treatment response was not associated with ADA TL, as R and NR showed similar concentrations (median [IQR] = 8.5 [3.9–14.8] µg/mL vs. 10.3 [7.9–14.5] µg/mL, respectively). The two patients that presented low ADA TL and AAA were both R.

**Fig. 3:**
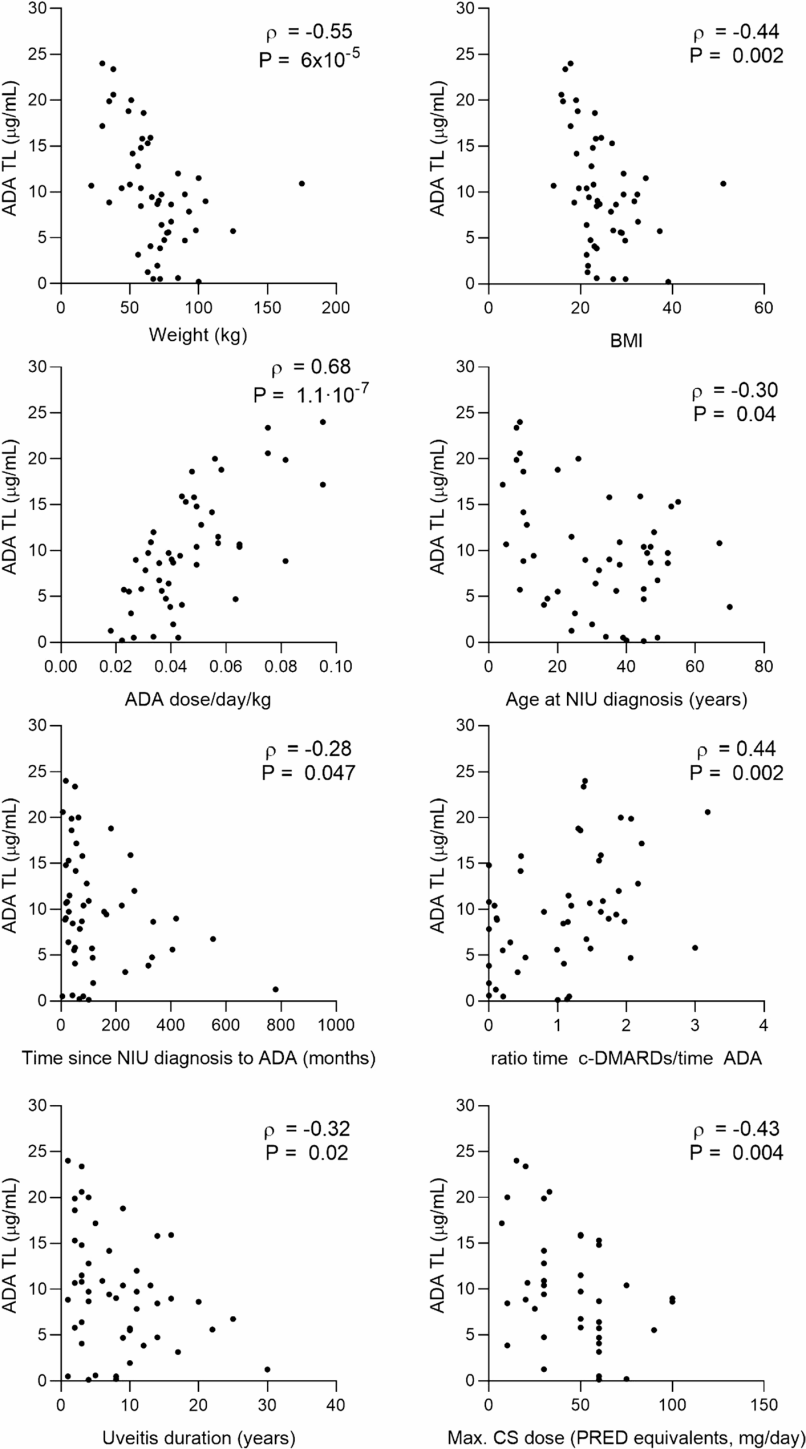
Correlation of ADA TL with clinical variables in patients with NIU

Significant correlations were found between ADA TL and clinical variables (Fig. 3). Negative correlations were found for weight and body mass index (BMI), age at uveitis diagnosis, uveitis duration, and time from diagnosis to ADA initiation. Maximum CS dose was also negatively correlated with ADA TL. A trend toward lower ADA TL was noted with longer ADA treatment duration (*ρ* = −0.25, *P* = 0.09). Positive correlations were observed with ADA dose (adjusted for administration interval and patient weight), year of uveitis diagnosis (*ρ* = 0.29, *P* = 0.04) and c-DMARD-related variables, including the number of current c-DMARDs (ρ = 0.43, *P* = 0.002) and the duration of concomitant ADA and c-DMARD therapy, both simultaneous *(ρ* = 0.42, *P* = 0.003) and non-simultaneous *(ρ* = 0.44, *P* = 0.002).

Further analysis showed that patients with high BMI (≥ 25) had significantly lower ADA TL compared to those with normal BMI (median [IQR] = 7.3 [5.3–10.0] µg/mL vs. 10.6 [6.0-17.6] µg/mL, *P* = 0.03). Patients on current c-DMARD therapy had significantly higher ADA TL than those who had discontinued c-DMARDs (median [IQR] = 10.9 [7.6–17.3] µg/mL vs. 6.0 [2.3–10.2] µg/mL, *P* = 0.003).

**Fig. 4:**
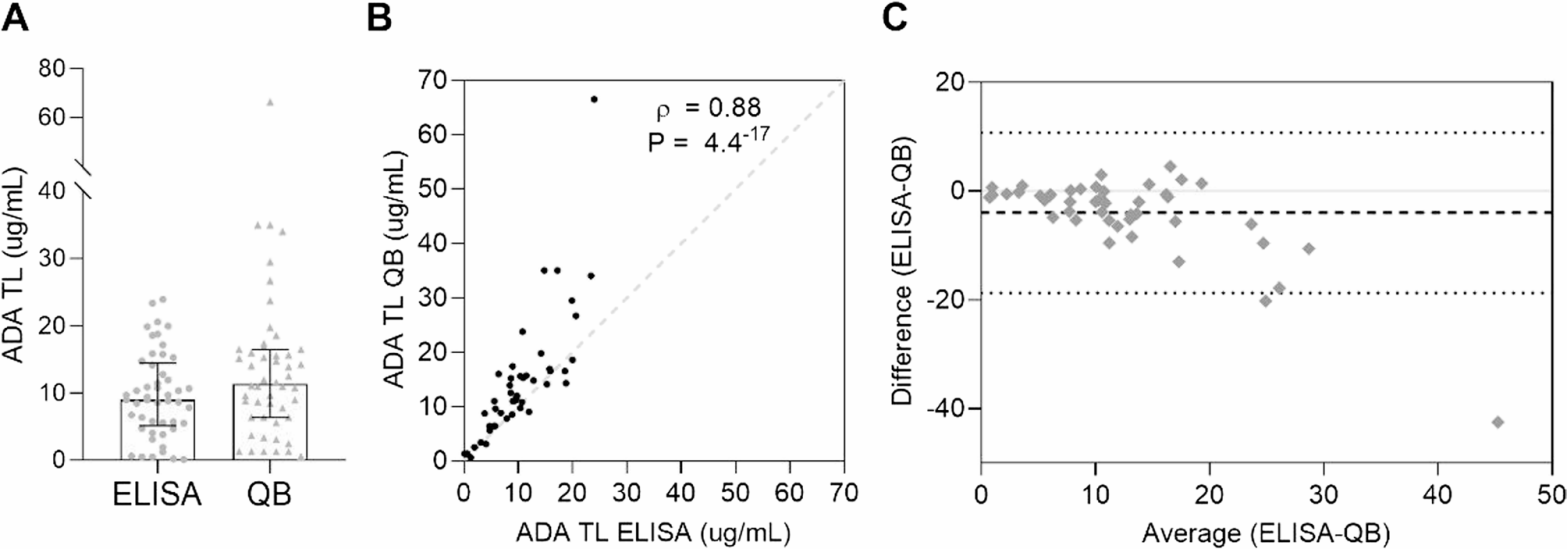
Comparison of the serological determination of ADA TL using Promonitor-ADL ELISA kit and Quantum Blue lateral flow immunoassay. ** A** ADA TL measured by ELISA and QB in patients with NIU. **B** Linear regression of ADA TL determined by both techniques. The bold line represents the best-fit line, the dashed lines represent the 95% confidence bands, and the light grey dashed line represents the identity line. **C ** Bland-Altman’s plot of the difference in ADL TL measured by ELISA and QB vs. the average (µg/mL) ADA TL values. The dashed line represents the mean bias and point lines represent the 95% limit of agreement

Serological ADA TL were measured using ELISA, the clinical gold standard, and with the QB point-of-care system (POC). QB levels were generally higher than those obtained by ELISA (Fig. 4A), but the difference was not statistically significant. Six patients had undetectable or very low (< 1.5 ug/mL) ADA TL by both methods, but only two had AAA. The drug concentration determined by both techniques was highly correlated (Fig. 4B), although agreement decreased at higher concentrations (Fig. 4C). All further analyses were performed with ADA TL determined by ELISA to adhere with the gold standard method in the clinic.

## Discusion

In the present cross-sectional study, a total of 91 eyes from 52 patients with NIU were analyzed across four Spanish tertiary-level hospitals, providing real-world evidence that prolonged use of ADA is safe, effective and common clinical practice for managing NIU. Based on the SUN criteria, 65.4% of patients were complete responders after a median treatment of 3.6 years, while 34.6% exhibited some degree of ocular inflammation. Although differences in methodology make the comparison of outcomes between studies difficult, these results align with prior real-world studies that consistently demonstrate the effectiveness of ADA in reducing ocular inflammation and enabling CS and IS tapering.

Cordero-Coma et al. [11] conducted a prospective study that included 25 patients, with a favorable clinical response in 72% of cases after 6 months of therapy, with 44% achieving complete remission. Raad et al. [20] carried out a retrospective cohort study that showed a significant reduction in ocular inflammation parameters from 64.1% of eyes before ADA initiation to 29.9% at 12 months, alongside sustained visual acuity improvement. While other studies such as that by Bitossi et al. reported ocular control rates of 80.4% within 12 months [21], their response criteria were less stringent than the SUN criteria applied in our study, which likely explains this difference.

Anterior uveitis was the most prevalent form in our cohort, contrasting with other reports, such as Raad et al. (34.7% panuveitis) [20] and Tang Lee Say et al. (45.7% panuveitis) [22]. Importantly, ADA proved effective across all uveitis subtypes in our cohort. This highlights ADA’s broader utility in clinical practice, despite the EMA restricting ADA indication to intermediate, posterior, and panuveitis. In fact, we observed a higher likelihood of clinical response in anterior uveitis. Regarding relapses, NR experienced significantly more intermediate relapses than R in our cohort, aligning with findings by Eurelings et al. [23], who reported that relapse-free survival in patients with intermediate NIU was shorter compared to those with other uveitis types. These findings, together with the observation that intermediate uveitis has the highest risk of NR (> 16-fold) in the present study, highlight the greater challenges in treating this subgroup of uveitis.

While long-term safety of ADA was excellent, mild-to-moderate, non-life-threatening self-reported AEs occurred in one-third of our patients, primarily infections, as has been previously described for anti-TNF therapy [17, 20]. The higher incidence reported in this study compared to previous reports [17, 20] likely reflects the longer exposure time to ADA, which allowed for more time to capture and report AEs.

Focus on bridging the gap between clinical outcomes and patient-reported experiences ensures a holistic approach that integrates clinical metrics and patient perspectives. Unlike other studies that focus solely on clinical response, our study also evaluates PROMs, providing a more comprehensive assessment of the treatment’s impact. In this study, only the general vision subscale of the VFQ-25 showed a higher score in R than NR, highlighting the pivotal role of vision in patients’ lives. However, it is crucial to note that the long-standing nature of NIU in our cohort likely played a role in these findings, as irreversible vision loss may have already occurred due to prior treatment delays or failures. This emphasizes the necessity of early and effective therapeutic interventions to preserve visual function and mitigate declines in QoL. Post hoc analyses of the controlled trials VISUAL-1 and VISUAL-2 have suggested statistically significant and clinically meaningful improvements in the NEI VFQ-25 with ADA compared with placebo [16]: ADA-treated patients with active uveitis (VISUAL-1) improved 11 of the 12 domains of the NEI VFQ-25, whereas 9 of the 12 domains were improved in the inactive NIU patients in VISUAL-2, with the most significant improvements seen in both studies in general vision and mental health. However, controlled trials findings not fully translate to real-world settings as CS or IS would have been used instead of placebo. Only one prospective, real-life cohort study has provided more pragmatic insights into the effectiveness of ADA in reducing the healthcare burden and improving QoL in NIU patients, with a median change of 4.7 (IQR, 0.4–14.4) in overall VFQ-25 score at 12 months vs. baseline (*p* < 0.0001) [17].

Concerning concomitant treatments, ADA allowed significant reductions in systemic CS, including complete discontinuation of oral CS. A recent report by the *International Ocular Inflammation Society (IOIS)* that collected data on real-world practice from 221 uveitis specialists across 53 countries, highlighted that prolonged use of CS should be minimized, favoring tapering to ≤ 10 mg/day or ideally ≤ 5 mg/day [24]. In the present study, the median CS dose was reduced to 5 mg/day, consistent with the drop in CS dose to 5.99 ± 6.5 mg/day after 6 months of ADA treatment reported by Corredores Dieb et al. [25], the reduction in CS dose to < 7.5 mg/day in > 95% of patients reported by Tang Lee Say et al. [22], and other previous real-world studies in both adult [17, 20, 23] and pediatric populations [26]. It is important to highlight that up to one-third of ophthalmologists prefer to maintain low-dose CS even after 6 months of therapy, aligning with recent rheumatological literature supporting better outcomes in patients receiving adjunctive low-dose of oral CS for 2 years [24]. Regarding immunosuppressive therapy, the reduction in c-DMARDs usage reported here (88.5–59.6%) was consistent with previous studies [17, 20, 27]. The most prescribed c-DMARD before and after ADA was MTX, in line with the *IOIS report* [24] where 84.0% of members indicated that combination of MTX and ADA was the most common systemic immunomodulatory regimen. Interestingly, recent real-world data from the AIDA registry in pediatric uveitis suggest that ADA monotherapy may be as effective as combination therapy with MTX in preventing relapses and preserving visual acuity, with similar drug retention and safety profiles over 36 months [28].

On the other hand, our findings support the equivalence of biosimilars to original ADA in efficacy and safety in the management of NIU. No significant differences were observed in clinical response rates, relapse rates, or AE profiles. These findings align with previous studies that found no significant differences in flare rates before and after switching from Humira to a biosimilar [29], as well as evidence of proven effectiveness of biosimilars in pediatric NIU [30].

The observed differences in response rates according to ADA dosing regimen are likely explained by clinical practice patterns. Patients with suboptimal control are more likely to have their regimen intensified to weekly administration, whereas those achieving sustained inactivity for usually at least 24 months are often tapered to extended dosing intervals prior to considering systemic immunomodulatory treatment withdrawal [24]. This selection process inherently concentrates more difficult-to-treat cases in the intensified group and stable, well-controlled patients in the extended interval group, potentially explaining the lower and higher response rates, respectively.

The persistence of NR (35%) underscores the need for further research into factors influencing treatment failure, including serum ADA TL. Monitoring serum ADA TL and detecting AAA has been suggested to optimize treatment in NIU, as some studies have reported a correlation between higher ADA TL and better clinical outcomes [11, 12]. However, biological drugs exhibit pharmacokinetic (PK) variability, influenced by factors such as anthropometry, drug absorption, immunogenicity, concomitant immunomodulators and genetics [31]. While higher BMI is a well-established factor influencing ADA PK [7], the association of c-DMARDs to higher ADA TL remains controversial [11, 23, 32, 33].

Although serum ADA TLs were influenced by BMI and c-DMARD use, their limited correlation with clinical response challenges their role as reliable therapeutic markers and highlights the challenge of defining optimal therapeutic targets. Comparisons with previous studies remain complex due to methodological differences in study design and response classification. In our cohort, patients with higher BMI (≥ 25) had lower ADA TL, whereas those under concomitant c-DMARD treatment showed higher ADA TL, suggesting that personalizing ADA doses by weight and maintaining concomitant c-DMARDs could help sustain serum ADA levels. However, no association was found between ADA TL and response rates. Reported therapeutic ranges in NIU vary widely, from ≥ 3.3 µg/mL, which has been linked to complete response in adults with a moderate 0.67 AUC [34], to 9.65–13 µg/mL associated with positive outcomes in children [33]. Notably, this second range [33] is much higher than those established for adults with RA (5–8 µg/mL) [9], IBD (8–12 µg/mL) [35], or psoriasis (3.51–7.00 µg/mL) [36]. Ocular barriers limiting ADA bioavailability may justify the need for higher systemic levels to achieve effective intraocular treatment [37]. These challenges are being addressed, particularly in preclinical studies [38], through alternative administration routes such as intravitreal injection, which could help bypass systemic exposure and enhance drug delivery to the target tissue.

Interestingly, we observed a trend towards decreasing ADA TL with longer treatment duration in our patients. Yuan et al., also reported a decline in ADA TL to zero at 12 months of tapering ADA regardless of relapse status [13], suggesting that serum levels may not be the primary determinant of ocular inflammation recurrence. This decline in serological ADA levels over time may result from various factors, including changes in immunogenicity, drug clearance, and clinical management decisions to taper doses. This further highlights the potential influence of treatment duration on ADA PK and patient outcomes, suggesting that the relevance of ADA TL as a monitoring tool may diminish over time.

Immunogenicity has been consistently associated with lower ADA TL and therefore therapy failure [23, 34], including a recent systematic review [32]. Notably, some studies distinguish between transient and permanent AAA [32]. While real-world studies report higher prevalence of AAA than clinical trials (9% − 35.7%) [12, 20, 23, 34, 39], this was not the case in our study, where only 2 patients (4%) presented AAA. Both patients showed very low or undetectable levels but achieved complete response, as previously described by Bellur et al. [34]. However, we were unable to explore the correlation between AAA presence, ADA TL, and disease activity. The mechanisms underlying AAA generation and the impact that immunogenicity fluctuation may have on treatment response are unclear [40], and the debate on the influence of concomitant immunosuppressive treatment on ADA TL and AAA development remains open [11, 23, 28, 32, 33]. This is particularly relevant in light of the aforementioned AIDA registry data [28], in which ADA monotherapy was as effective as combination therapy with MTX, challenging the notion that MTX is essential to sustain ADA efficacy.

Our data suggest that ADA efficacy is not solely dose-dependent but also time-dependent and may be influenced by a myriad of patient-specific factors, including genetics, immunogenicity, disease chronicity and prior or concomitant treatments. This reinforces the importance of tailoring treatment to individual patient profiles rather than applying generalized therapeutic protocols. Current TDM practices in NIU, particularly regarding timing, cost-effectiveness, and clinical thresholds remain insufficient, even though we believe that for cases of primary or secondary loss of response TDM may still hold value as a complementary tool when integrated with other biomarkers and patient-specific data. However, the limited association between ADA TL and response suggests that blood-derived biomarkers alone may not be sufficient to guide treatment adjustments. Novel multi-omic approaches using ocular fluids could provide a more accurate representation of the underlying biological processes, as demonstrated in a subset of patients from this cohort, where differential tear proteomic profiles were identified through non-invasive tear collection that allowed for response stratification [41].

Limitations of this study include its cross-sectional design, which prevents the longitudinal evaluation of treatment retention or disease progression and limits the ability to assess the time needed for complete resolution of intraocular inflammation in different uveitis subtypes. This is particularly relevant in recalcitrant forms of intermediate, posterior uveitis and retinal vasculitis, where inflammatory control may take substantially longer than in anterior uveitis. The low prevalence and heterogeneous clinical behavior of NIU also make designing an adequately powered sample challenging. Furthermore, variability in biosimilar usage and treatment duration complicates the comparison of drug levels and outcomes. Further real-world studies, ideally using prospective cohort designs, are needed to define the timing and clinical thresholds for TDM in NIU, as well as to evaluate the cost-effectiveness of monitoring serum ADA TL and AAAs.

## Conclusions

Our findings confirm that ADA demonstrates sustained long-term effectiveness and safety in managing refractory NIU, significantly reducing the need for systemic CS and c-DMARDs. Original ADA and biosimilars show comparable effectiveness and safety. Although PROMs were not the primary focus of this study, our findings suggest that vision-related QoL improvements were mostly driven by general vision perception, rather than other objective parameters of intraocular inflamation. Moreover, this study highlights that while serum ADA levels were influenced by BMI and c-DMARD use, they did not correlate with clinical response, suggesting that the utility of therapeutic drug monitoring in NIU may be limited over time.

## Data Availability

The datasets used and/or analysed during the current study are not publicly available due to patient confidentiality and institutional restrictions but are available from the corresponding author on reasonable request.

## Abbreviations

AAA: Anti-adalimumab antibodies
ACC: Anterior chamber cells
ADA: Adalimumab
ADA TL: Adalimumab trough levels
AE: Adverse event
BCVA: Best corrected visual acuity
BMI: Body mass index
c-DMARD: Conventional disease-modifying antirheumatic drug
CS: Corticosteroid
ERM: Epiretinal membrane
HRQoL: Health-related quality of life
IBD: Inflammatory bowel disease
IOIS: International Ocular Inflammation Society
IQR: Interquartile range
JIA: Juvenile idiopathic arthritis
logMAR: Logarithm of the minimum angle of resolution
ME: Macular edema
MNV: Macular neovascularization
MTX: Methotrexate
NEI: National Eye Institute
NEI-VFQ-25: National Eye Institute Visual Function Questionnaire-25
NIU: Non-infectious uveitis
NR: Non-responder
OCT: Optical coherence tomography
PK: Pharmacokinetics
POC: Point-of-care system
PREMs: Patient-reported experience measures
PROM: Patient-reported outcome measure
QB: Quantum Blue
QoL: Quality of life
R: Responder
RA: Rheumatoid arthritis
REDCap: Research Electronic Data Capture
SD: Standard deviation
SUN: Standardization of Uveitis Nomenclature
TDM: Therapeutic drug monitoring
VH: Vitreous haze grading
VRQoL: Vision-related quality of life
